# 4-[(1*E*)-3-(2,6-Dichloro-3-fluoro­phen­yl)-3-oxoprop-1-en-1-yl]benzonitrile

**DOI:** 10.1107/S1600536812015589

**Published:** 2012-04-18

**Authors:** Aletti S. Praveen, Hemmige S. Yathirajan, Badiadka Narayana, Thomas Gerber, Eric Hosten, Richard Betz

**Affiliations:** a University of Mysore, Department of Studies in Chemistry, Manasagangotri, Mysore 570 006, India; b Mangalore University, Department of Studies in Chemistry, Mangalagangotri 574 199, India; c Nelson Mandela Metropolitan University, Summerstrand Campus, Department of Chemistry, University Way, Summerstrand, PO Box 77000, Port Elizabeth 6031, South Africa

## Abstract

In the title mol­ecule, C_16_H_8_Cl_2_FNO, the benzene rings form a dihedral angle of 78.69 (8)°. The F atom is disordered over two positions in a 0.530 (3):0.470 (3) ratio. The crystal packing exhibits π–π inter­actions between dichloro-substituted rings [centroid–centroid distance = 3.6671 (10) Å] and weak inter­molecular C—H⋯F contacts.

## Related literature

For the biological activity of chalcones, see: Rajendra Prasad *et al.* (2008 ▶); Shivakumar *et al.* (2005 ▶); Churkin *et al.* (1982 ▶); Herencia *et al.* (1998 ▶). For a related structure, see: Betz *et al.* (2012 ▶). For the graph-set analysis of hydrogen bonds, see: Etter *et al.* (1990 ▶); Bernstein *et al.* (1995 ▶).
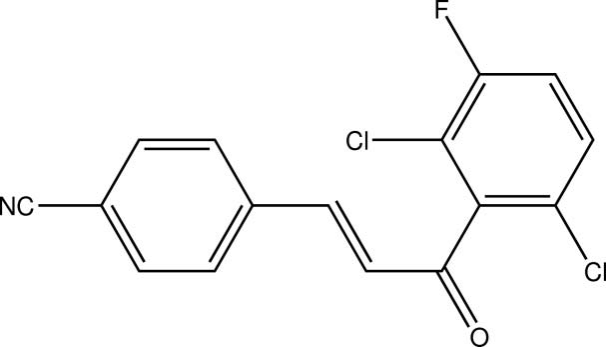


## Experimental

### 

#### Crystal data

C_16_H_8_Cl_2_FNO
*M* *_r_* = 320.13
Monoclinic, 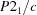

*a* = 13.2751 (3) Å
*b* = 8.5002 (2) Å
*c* = 13.9854 (3) Å
β = 116.773 (1)°
*V* = 1408.95 (6) Å^3^
*Z* = 4
Mo *K*α radiation
μ = 0.47 mm^−1^
*T* = 200 K
0.55 × 0.32 × 0.20 mm

#### Data collection

Bruker APEXII CCD diffractometer
Absorption correction: multi-scan (*SADABS*; Bruker, 2008 ▶) *T*_min_ = 0.783, *T*_max_ = 0.913
12885 measured reflections
3480 independent reflections
2904 reflections with *I* > 2σ(*I*)
*R*_int_ = 0.013

#### Refinement

*R*[*F* ^2^ > 2σ(*F* ^2^)] = 0.037
*wR*(*F* ^2^) = 0.098
*S* = 1.05
3480 reflections
200 parameters
H-atom parameters constrained
Δρ_max_ = 0.41 e Å^−3^
Δρ_min_ = −0.43 e Å^−3^

### 

Data collection: *APEX2* (Bruker, 2010 ▶); cell refinement: *SAINT* (Bruker, 2010 ▶); data reduction: *SAINT*; program(s) used to solve structure: *SHELXS97* (Sheldrick, 2008 ▶); program(s) used to refine structure: *SHELXL97* (Sheldrick, 2008 ▶); molecular graphics: *ORTEP-3* (Farrugia, 1997 ▶) and *Mercury* (Macrae *et al.*, 2008 ▶); software used to prepare material for publication: *SHELXL97* and *PLATON* (Spek, 2009 ▶).

## Supplementary Material

Crystal structure: contains datablock(s) I, global. DOI: 10.1107/S1600536812015589/cv5282sup1.cif

Supplementary material file. DOI: 10.1107/S1600536812015589/cv5282Isup2.cdx

Structure factors: contains datablock(s) I. DOI: 10.1107/S1600536812015589/cv5282Isup3.hkl

Supplementary material file. DOI: 10.1107/S1600536812015589/cv5282Isup4.cml

Additional supplementary materials:  crystallographic information; 3D view; checkCIF report

## Figures and Tables

**Table 1:** Hydrogen-bond geometry (Å, °)

*D*—H⋯*A*	*D*—H	H⋯*A*	*D*⋯*A*	*D*—H⋯*A*
C1—H1⋯F1*A*^i^	0.95	2.50	3.130 (2)	124

Symmetry code: (i) 
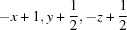
.

**Fig. 1.**
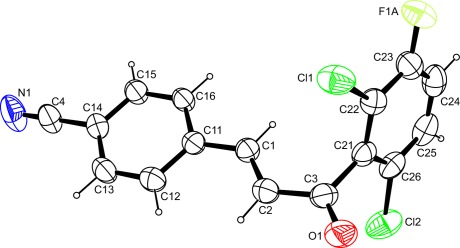
The molecular structure of the title compound showing the atomic numbering and 50% probability displacement ellipsoids. Only the major part of the disordered fluorine atom is shown.

**Fig. 2.**
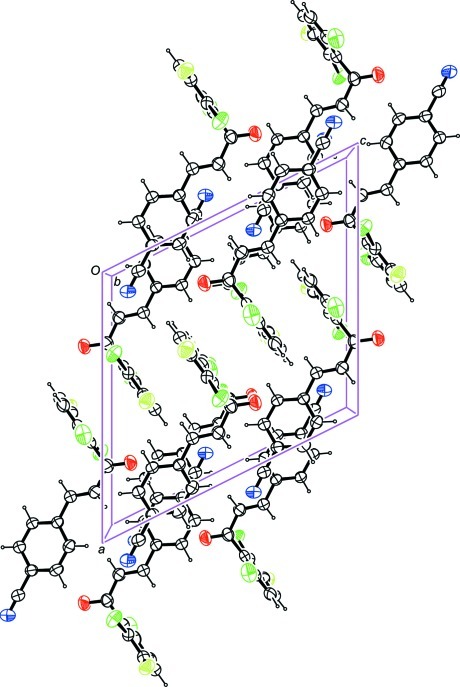
A portion of the crystal packing viewed along [010].

## supplementary crystallographic information

### Comment

Chalcones are the products of condensation reactions of aromatic aldehydes with acetophenones in the presence of alkali. Chalcones constitute an important group of natural products, and some of them possess a wide range of biological activities such as antimicrobial (Rajendra Prasad *et al.*, 2008), antitubercular (Shivakumar *et al.*, 2005), antiviral (Churkin *et al.*, 1982) and anti-inflammatory activity (Herencia *et al.*, 1998). The crystal structures of some chalcones such as have been reported in the literature. As a part of our ongoing studies on chalcones, the title compound (I) has been synthesized and characterized by X-ray diffraction.

In (I) (Fig. 1), all bond lengths and angles are normal and correspond to those observed in (2*E*)-1-(2,6-dichloro-3-fluorophenyl)-3-(4-fluorophenyl)prop-2-en-1-one (Betz *et al.*, 2012). The fluorine atom is disordered over two positions with site occupancy factors of 0.530 (3) and 0.470 (3), respectively. The mean planes of the two aromatic rings for a dihedral angle of 78.69 (8)°.

In the crystal, C–H···F contacts (Table 1) whose range falls by more than 0.1 Å below the sum of van-der-Waals radii of the respective atoms are present. These are supported by one of the vinylic hydrogen atoms as well as the disordered fluorine atom. In terms of graph-set analysis (Etter *et al.*, 1990; Bernstein *et al.*, 1995), the descriptor for these hydrogen bonds is *C*^1^_1_(8) on the unary level. The shortest intercentroid distance between two aromatic systems was found at 3.6674 (10) Å and is apparent between the halogenated phenyl rings. The packing of the title compound in the crystal structure is shown in Figure 2.

### Experimental

To a stirred solution of 1-(2,6-dichloro-3-fluorophenyl)ethanone (1 g, 4.8 mmol) and 4-formylbenzonitrile (0.62 g, 4.8 mmol) in ethanol (10 ml), powdered KOH (0.40 g, 7.2 mmol) was added at 273 K. The reaction mixture was stirred at room temperature for 2 h. After completion of the reaction, the mixture was poured into ice cold water and subsequently acidified with 1.5 N HCl (pH ~3). The precipitated solid was filtered and dried to afford 1 g of the title compound as pale yellow solid in 91% yield. The single-crystal was grown from a mixture of toluene:acetone (*v*:*v* = 1:1) by slow evaporation at room temperature (m.p.: 414–417 K).

### Refinement

C-bound H atoms were placed in calculated positions (C—H 0.95 Å) and were included in the refinement in the riding model approximation, with *U*(H) set to 1.2*U*_eq_(C).

### Crystal data

**Table:**

C_16_H_8_Cl_2_FNO	*F*(000) = 648
*M**_r_* = 320.13	*D*_x_ = 1.509 Mg m^−^^3^
Monoclinic, *P*2_1_/*c*	Melting point = 414–417 K
Hall symbol: -P 2ybc	Mo *K*α radiation, λ = 0.71073 Å
*a* = 13.2751 (3) Å	Cell parameters from 7660 reflections
*b* = 8.5002 (2) Å	θ = 2.9–28.3°
*c* = 13.9854 (3) Å	µ = 0.47 mm^−^^1^
β = 116.773 (1)°	*T* = 200 K
*V* = 1408.95 (6) Å^3^	Platelet, green
*Z* = 4	0.55 × 0.32 × 0.20 mm

### Data collection

**Table:**

Bruker APEXII CCD diffractometer	3480 independent reflections
Radiation source: fine-focus sealed tube	2904 reflections with *I* > 2σ(*I*)
Graphite monochromator	*R*_int_ = 0.013
φ and ω scans	θ_max_ = 28.3°, θ_min_ = 2.9°
Absorption correction: multi-scan (*SADABS*; Bruker, 2008)	*h* = −17→17
*T*_min_ = 0.783, *T*_max_ = 0.913	*k* = −8→11
12885 measured reflections	*l* = −18→18

### Refinement

**Table:**

Refinement on *F*^2^	Primary atom site location: structure-invariant direct methods
Least-squares matrix: full	Secondary atom site location: difference Fourier map
*R*[*F*^2^ > 2σ(*F*^2^)] = 0.037	Hydrogen site location: inferred from neighbouring sites
*wR*(*F*^2^) = 0.098	H-atom parameters constrained
*S* = 1.05	*w* = 1/[σ^2^(*F*_o_^2^) + (0.0358*P*)^2^ + 0.6287*P*] where *P* = (*F*_o_^2^ + 2*F*_c_^2^)/3
3480 reflections	(Δ/σ)_max_ < 0.001
200 parameters	Δρ_max_ = 0.41 e Å^−^^3^
0 restraints	Δρ_min_ = −0.43 e Å^−^^3^

### Fractional atomic coordinates and isotropic or equivalent isotropic displacement parameters (Å^2^)

**Table:**

	*x*	*y*	*z*	*U*_iso_*/*U*_eq_	Occ. (<1)
Cl1	0.32198 (4)	0.25664 (5)	0.03764 (4)	0.05826 (14)	
Cl2	0.43426 (5)	0.87196 (6)	0.08058 (4)	0.06998 (17)	
O1	0.23229 (11)	0.62352 (16)	−0.09939 (9)	0.0582 (3)	
N1	−0.11644 (13)	0.5937 (3)	0.40221 (14)	0.0785 (6)	
C1	0.21695 (12)	0.57997 (17)	0.14653 (12)	0.0392 (3)	
H1	0.2927	0.5473	0.1891	0.047*	
C2	0.18838 (14)	0.6169 (2)	0.04481 (13)	0.0460 (4)	
H2	0.1140	0.6544	0.0017	0.055*	
C3	0.26474 (14)	0.60326 (19)	−0.00432 (12)	0.0435 (3)	
C4	−0.06074 (13)	0.5902 (2)	0.35969 (14)	0.0525 (4)	
C11	0.14352 (12)	0.58457 (17)	0.19935 (11)	0.0370 (3)	
C12	0.03215 (13)	0.6397 (2)	0.14813 (13)	0.0487 (4)	
H12	0.0022	0.6771	0.0765	0.058*	
C13	−0.03410 (13)	0.6403 (2)	0.20037 (13)	0.0489 (4)	
H13	−0.1097	0.6771	0.1647	0.059*	
C14	0.00933 (12)	0.58722 (18)	0.30543 (12)	0.0408 (3)	
C15	0.11978 (12)	0.5340 (2)	0.35809 (12)	0.0440 (3)	
H15	0.1498	0.4984	0.4301	0.053*	
C16	0.18571 (12)	0.53320 (19)	0.30467 (12)	0.0407 (3)	
H16	0.2614	0.4968	0.3407	0.049*	
C21	0.38739 (13)	0.56076 (18)	0.06658 (11)	0.0394 (3)	
C22	0.42114 (12)	0.40474 (19)	0.08897 (11)	0.0404 (3)	
C24	0.61343 (14)	0.4808 (3)	0.19589 (13)	0.0538 (4)	
H24	0.6899	0.4539	0.2409	0.065*	
C26	0.47029 (15)	0.6754 (2)	0.10883 (13)	0.0467 (4)	
C23	0.53293 (14)	0.3657 (2)	0.15222 (13)	0.0474 (4)	
H23	0.5542	0.2582	0.1656	0.057*	0.470 (3)
F1B	0.6550 (2)	0.7502 (4)	0.2076 (2)	0.0847 (11)	0.470 (3)
C25	0.58158 (15)	0.6354 (2)	0.17347 (14)	0.0545 (4)	
H25	0.6367	0.7160	0.2027	0.065*	0.530 (3)
F1A	0.56085 (16)	0.2148 (3)	0.16948 (16)	0.0610 (7)	0.530 (3)

### Atomic displacement parameters (Å^2^)

**Table:**

	*U*^11^	*U*^22^	*U*^33^	*U*^12^	*U*^13^	*U*^23^
Cl1	0.0520 (2)	0.0417 (2)	0.0616 (3)	−0.00337 (17)	0.00832 (19)	0.00136 (18)
Cl2	0.1033 (4)	0.0436 (2)	0.0648 (3)	−0.0126 (2)	0.0394 (3)	−0.0003 (2)
O1	0.0635 (7)	0.0718 (9)	0.0403 (6)	0.0168 (6)	0.0242 (6)	0.0145 (6)
N1	0.0455 (8)	0.1321 (18)	0.0629 (10)	0.0129 (10)	0.0288 (8)	−0.0008 (11)
C1	0.0376 (7)	0.0386 (7)	0.0407 (7)	0.0002 (6)	0.0171 (6)	0.0005 (6)
C2	0.0444 (8)	0.0502 (9)	0.0432 (8)	0.0089 (7)	0.0195 (7)	0.0076 (7)
C3	0.0508 (8)	0.0405 (8)	0.0406 (8)	0.0047 (7)	0.0217 (7)	0.0052 (6)
C4	0.0361 (7)	0.0725 (12)	0.0473 (9)	0.0051 (8)	0.0172 (7)	−0.0027 (8)
C11	0.0355 (7)	0.0349 (7)	0.0389 (7)	−0.0007 (6)	0.0153 (6)	−0.0010 (6)
C12	0.0397 (8)	0.0596 (10)	0.0418 (8)	0.0076 (7)	0.0139 (6)	0.0099 (7)
C13	0.0336 (7)	0.0586 (10)	0.0497 (9)	0.0088 (7)	0.0146 (6)	0.0058 (7)
C14	0.0346 (7)	0.0428 (8)	0.0451 (8)	−0.0006 (6)	0.0182 (6)	−0.0052 (6)
C15	0.0392 (7)	0.0525 (9)	0.0404 (7)	0.0054 (6)	0.0179 (6)	0.0025 (7)
C16	0.0332 (7)	0.0465 (8)	0.0401 (7)	0.0059 (6)	0.0147 (6)	0.0024 (6)
C21	0.0461 (8)	0.0440 (8)	0.0327 (7)	0.0000 (6)	0.0219 (6)	0.0015 (6)
C22	0.0405 (7)	0.0443 (8)	0.0348 (7)	−0.0011 (6)	0.0155 (6)	−0.0012 (6)
C24	0.0390 (8)	0.0831 (13)	0.0393 (8)	−0.0030 (8)	0.0176 (7)	−0.0014 (8)
C26	0.0603 (9)	0.0472 (9)	0.0411 (8)	−0.0065 (7)	0.0303 (7)	−0.0005 (7)
C23	0.0464 (8)	0.0571 (10)	0.0389 (7)	0.0080 (7)	0.0193 (7)	0.0015 (7)
F1B	0.0738 (17)	0.107 (2)	0.0622 (16)	−0.0519 (16)	0.0211 (13)	−0.0060 (14)
C25	0.0535 (9)	0.0717 (12)	0.0433 (8)	−0.0211 (9)	0.0260 (8)	−0.0071 (8)
F1A	0.0529 (11)	0.0588 (13)	0.0594 (12)	0.0184 (9)	0.0147 (9)	0.0096 (9)

### Geometric parameters (Å, º)

**Table:**

Cl1—C22	1.7267 (16)	C13—H13	0.9500
Cl2—C26	1.7338 (18)	C14—C15	1.387 (2)
O1—C3	1.2112 (18)	C15—C16	1.383 (2)
N1—C4	1.139 (2)	C15—H15	0.9500
C1—C2	1.334 (2)	C16—H16	0.9500
C1—C11	1.465 (2)	C21—C26	1.387 (2)
C1—H1	0.9500	C21—C22	1.390 (2)
C2—C3	1.463 (2)	C22—C23	1.383 (2)
C2—H2	0.9500	C24—C23	1.373 (3)
C3—C21	1.520 (2)	C24—C25	1.373 (3)
C4—C14	1.441 (2)	C24—H24	0.9500
C11—C16	1.390 (2)	C26—C25	1.383 (3)
C11—C12	1.402 (2)	C23—F1A	1.327 (3)
C12—C13	1.373 (2)	C23—H23	0.9500
C12—H12	0.9500	F1B—C25	1.308 (3)
C13—C14	1.390 (2)	C25—H25	0.9500
			
C2—C1—C11	126.67 (14)	C15—C16—H16	119.3
C2—C1—H1	116.7	C11—C16—H16	119.3
C11—C1—H1	116.7	C26—C21—C22	117.46 (14)
C1—C2—C3	123.90 (14)	C26—C21—C3	121.47 (15)
C1—C2—H2	118.1	C22—C21—C3	121.05 (14)
C3—C2—H2	118.1	C23—C22—C21	121.15 (15)
O1—C3—C2	121.68 (15)	C23—C22—Cl1	119.24 (13)
O1—C3—C21	119.79 (14)	C21—C22—Cl1	119.61 (11)
C2—C3—C21	118.53 (13)	C23—C24—C25	118.87 (16)
N1—C4—C14	179.4 (2)	C23—C24—H24	120.6
C16—C11—C12	118.39 (14)	C25—C24—H24	120.6
C16—C11—C1	118.96 (13)	C25—C26—C21	120.98 (17)
C12—C11—C1	122.65 (13)	C25—C26—Cl2	119.32 (14)
C13—C12—C11	120.68 (15)	C21—C26—Cl2	119.70 (13)
C13—C12—H12	119.7	F1A—C23—C24	120.69 (17)
C11—C12—H12	119.7	F1A—C23—C22	118.67 (17)
C12—C13—C14	120.06 (14)	C24—C23—C22	120.64 (17)
C12—C13—H13	120.0	F1A—C23—H23	1.0
C14—C13—H13	120.0	C24—C23—H23	119.7
C15—C14—C13	120.21 (14)	C22—C23—H23	119.7
C15—C14—C4	120.30 (14)	F1B—C25—C24	121.9 (2)
C13—C14—C4	119.48 (14)	F1B—C25—C26	117.1 (2)
C16—C15—C14	119.30 (14)	C24—C25—C26	120.87 (16)
C16—C15—H15	120.4	F1B—C25—H25	3.7
C14—C15—H15	120.4	C24—C25—H25	119.6
C15—C16—C11	121.34 (13)	C26—C25—H25	119.6
			
C11—C1—C2—C3	−177.25 (15)	C26—C21—C22—C23	0.9 (2)
C1—C2—C3—O1	172.76 (17)	C3—C21—C22—C23	179.07 (13)
C1—C2—C3—C21	−6.6 (2)	C26—C21—C22—Cl1	−179.43 (11)
C2—C1—C11—C16	176.31 (16)	C3—C21—C22—Cl1	−1.24 (19)
C2—C1—C11—C12	−4.1 (3)	C22—C21—C26—C25	−2.0 (2)
C16—C11—C12—C13	−1.1 (2)	C3—C21—C26—C25	179.84 (14)
C1—C11—C12—C13	179.29 (16)	C22—C21—C26—Cl2	178.12 (11)
C11—C12—C13—C14	0.6 (3)	C3—C21—C26—Cl2	−0.07 (19)
C12—C13—C14—C15	0.2 (3)	C25—C24—C23—F1A	178.08 (18)
C12—C13—C14—C4	179.32 (17)	C25—C24—C23—C22	−1.7 (2)
N1—C4—C14—C15	136 (21)	C21—C22—C23—F1A	−178.81 (16)
N1—C4—C14—C13	−43 (21)	Cl1—C22—C23—F1A	1.5 (2)
C13—C14—C15—C16	−0.5 (2)	C21—C22—C23—C24	0.9 (2)
C4—C14—C15—C16	−179.57 (16)	Cl1—C22—C23—C24	−178.75 (12)
C14—C15—C16—C11	0.0 (2)	C23—C24—C25—F1B	−176.2 (2)
C12—C11—C16—C15	0.8 (2)	C23—C24—C25—C26	0.6 (2)
C1—C11—C16—C15	−179.54 (14)	C21—C26—C25—F1B	178.18 (19)
O1—C3—C21—C26	84.6 (2)	Cl2—C26—C25—F1B	−1.9 (2)
C2—C3—C21—C26	−96.08 (18)	C21—C26—C25—C24	1.3 (2)
O1—C3—C21—C22	−93.55 (19)	Cl2—C26—C25—C24	−178.81 (13)
C2—C3—C21—C22	85.81 (19)		

### Hydrogen-bond geometry (Å, º)

**Table:**

*D*—H···*A*	*D*—H	H···*A*	*D*···*A*	*D*—H···*A*
C1—H1···F1*A*^i^	0.95	2.50	3.130 (2)	124

Symmetry code: (i) −*x*+1, *y*+1/2, −*z*+1/2.

## References

[bb1] Bernstein, J., Davis, R. E., Shimoni, L. & Chang, N.-L. (1995). *Angew. Chem. Int. Ed. Engl.* **34**, 1555–1573.

[bb2] Betz, R., Gerber, T., Hosten, E., Praveen, A. S., Yathirajan, H. S. & Narayana, B. (2012). *Acta Cryst.* E**68**, o512. 10.1107/S1600536812002589 PMC3275256 22347112

[bb3] Bruker (2008). *SADABS* Bruker Inc., Madison, Wisconsin, USA.

[bb4] Bruker (2010). *APEX2* and *SAINT* Bruker AXS Inc., Madison, Wisconsin, USA.

[bb5] Churkin, Yu. D., Panfilova, L. V., Boreko, E. I., Timofeeva, M. M. & Votyakov, V. I. (1982). *Pharm. Chem. J.* **16**, 103–105.

[bb6] Etter, M. C., MacDonald, J. C. & Bernstein, J. (1990). *Acta Cryst.* B**46**, 256–262. 10.1107/s0108768189012929 2344397

[bb7] Farrugia, L. J. (1997). *J. Appl. Cryst.* **30**, 565.

[bb8] Herencia, F., Ferrandiz, M. L., Ubeda, A., Dominguez, J., Charris, J. E., Lobo, G. M. & Alcaraz, M. J. (1998). *Bioorg. Med. Chem. Lett.* **8**, 1169–1174. 10.1016/s0960-894x(98)00179-6 9871729

[bb9] Macrae, C. F., Bruno, I. J., Chisholm, J. A., Edgington, P. R., McCabe, P., Pidcock, E., Rodriguez-Monge, L., Taylor, R., van de Streek, J. & Wood, P. A. (2008). *J. Appl. Cryst.* **41**, 466–470.

[bb10] Rajendra Prasad, Y., Praveen Kumar, P., Ravi Kumar, P. & Srinivas Rao, A. (2008). *E-J. Chem.* **5**, 144–148.

[bb11] Sheldrick, G. M. (2008). *Acta Cryst.* A**64**, 112–122. 10.1107/S0108767307043930 18156677

[bb12] Shivakumar, P. M., Geetha Babu, S. M. & Mukesh, D. (2005). *Chem. Pharm. Bull.* **55**, 44–49. 10.1248/cpb.55.44 17202700

[bb13] Spek, A. L. (2009). *Acta Cryst.* D**65**, 148–155. 10.1107/S090744490804362X PMC2631630 19171970

